# Effect of interventions for the management of sleep disturbances in patients with long COVID: a systematic review and meta-analysis of randomized controlled trials

**DOI:** 10.5664/jcsm.11782

**Published:** 2025-11-01

**Authors:** Dai Yi Goh, Wai Ching Lam, Linda L. D. Zhong

**Affiliations:** ^1^ School of Biological Sciences, Nanyang Technological University, Singapore; ^2^ Department of Epidemiology, Harvard T.H. Chan School of Public Health, Boston, Massachusetts; ^3^ Chinese Medicine Teaching and Research Division, School of Chinese Medicine, Hong Kong Baptist University, Hong Kong SAR, China; ^4^ Hong Kong Chinese Medicine Clinical Study Centre, School of Chinese Medicine, Hong Kong Baptist University, Hong Kong SAR, China

**Keywords:** long COVID, interventions, treatments, sleep disturbances, systematic review, meta-analysis

## Abstract

**Study Objectives:**

Long COVID presents with symptoms that persist for weeks or months postinfection, with sleep disturbances that significantly affect quality of life. The diverse approaches to managing sleep disturbances highlight the need for comparing treatment effectiveness to improve patient outcomes. This study systematically reviews and conducts a meta-analysis of randomized controlled trials to assess the effectiveness of current interventions for sleep disturbances in patients with long COVID and explores the underlying mechanisms and promising treatments.

**Methods:**

Relevant studies were identified through a comprehensive literature search across Embase, Web of Science, PubMed, Cochrane Library, China National Knowledge Infrastructure, and Wanfang Data databases. The included studies focused on interventions aimed at managing patients with long COVID with sleep disturbances. Data extraction and analysis were performed, followed by a meta-analysis of comparable studies. The quality of evidence was assessed using the Cochrane Risk of Bias Tool (RoB 2.0) and the Grading of Recommendations, Assessment, Development, and Evaluation system.

**Results:**

Out of 3,352 retrieved studies, 14 were included in the systematic review and 2 in the meta-analysis. Interventions were categorized as pharmacological and nonpharmacological. Whereas most studies indicated improved sleep quality measured by standardized scales, some did not demonstrate significant benefits. The quality of evidence varied from low to moderate.

**Conclusions:**

The results suggest that sleep disturbances in patients with long COVID result from a complex interplay of physiological, psychological, and neurological factors. Both pharmacological and nonpharmacological interventions show potential in managing these disturbances, with nonpharmacological approaches showing particular promise. To establish more robust evidence, more high-quality, large-scale randomized controlled trials are necessary in future research.

**Citation:**

Goh DY, Lam WC, Zhong LLD. Effect of interventions for the management of sleep disturbances in patients with long COVID: a systematic review and meta-analysis of randomized controlled trials. *J Clin Sleep Med.* 2025;21(11):1993–2005.

**BRIEF SUMMARY**

**Current Knowledge/Study Rationale:** Sleep disturbances are a prevalent symptom of long COVID, significantly impacting patients’ quality of life. This study aimed to comprehensively assess the effectiveness of both pharmacological and nonpharmacological interventions, addressing the current lack of standardized treatment options and systematic evaluations.

**Study Impact:** The findings of the study underscore the promise of therapeutic exercises and natural remedies as effective, low-risk interventions for managing sleep disturbances in long COVID. By offering insights into tailored treatment approaches, it lays a foundation for future research to refine care strategies, improve clinical outcomes, enhance recovery, and guide the development of evidence-based therapeutic protocols for this complex condition.

## INTRODUCTION

Long COVID is a complex condition characterized by more than 200 documented symptoms^1^ that persist or develop following an initial SARS-CoV-2 infection.^2^ This multisystemic disease affects various bodily functions and causes significant neurological and psychological distress.^1^ According to the World Health Organization, 10–20% of COVID-19 patients go on to develop long COVID.^3^ With over 65 million cases reported worldwide as of 2023,^4^ long COVID presents challenges across all demographics, with particular prevalence in females and adults aged 18 years and older,^5^ regardless of the initial infection’s severity.^1^^,^^6^

Long COVID has many definitions. According to the Centers for Disease Control and Prevention, patients with long COVID experience a “range of ongoing symptoms and conditions” lasting at least 3 months postinfection.^2^ The National Center for Infectious Diseases describes it as a group of symptoms in recovered COVID-19 patients that “persist or emerge four weeks after the initial infection.”^7^ The National Institute for Health and Care Excellence further defines long COVID as symptoms developing during or after infection that prolong for “more than 12 weeks” and cannot be explained by an alternative diagnosis.^8^ Additionally, the World Health Organization characterizes long COVID as the persistence or emergence of new symptoms 3 months after initial infection, lasting at least 2 months without any other explanation.^3^

Among the neurological and psychological symptoms reported, sleep disturbances are particularly prevalent.^9^ Sleep disturbances encompass disorders that disrupt normal sleep patterns, classified in the *International Classification of Sleep Disorders*, third edition into 6 major categories: insomnia, sleep-related breathing disorders, hypersomnolence and narcolepsy, circadian rhythm disorders, parasomnias, and sleep-related movement disorders.^10^ Studies on long COVID–associated sleep disturbances have identified common issues such as insomnia, frequent awakenings,^11^ difficulty falling or staying asleep,^12^ and excessive daytime sleepiness.^13^ Chinvararak and Chalder^9^ found that about 46% of patients with long COVID experienced sleep disturbances, with insomnia affecting 38%. Similarly, Seighali et al’s^14^ meta-analysis reported a 45% prevalence of sleep disorders among 15,362 patients. If not adequately managed, these disturbances can impair physical, psychological and social health,^15^ increase anxiety^16^ and dyspnoea,^11^ and significantly reduce quality of life.^16^

Despite the high prevalence of sleep disturbances in patients with long COVID, their underlying mechanisms remain unclear. It is believed to involve a complex interplay of physiological, psychological, and neurological factors. The initial SARS-CoV-2 infection may trigger inflammatory responses that disrupt neurotransmitter systems essential for sleep regulation. Co-occurring symptoms such as fatigue and anxiety often accompany these disturbances,^17^ whereby psychological factors, such as stress and anxiety about the illness,^18^ and fear of social isolation can further worsen sleep disturbances.^12^ Supporting this, Sunada et al^18^ noted significantly higher levels of plasma adrenocorticotropin, a stress hormone, in patients with long COVID experiencing sleep disturbances.

Similar postviral syndromes, such as chronic post-SARS conditions, have been associated with prolonged neurocognitive and sleep-related symptoms. In managing post-SARS conditions, multidisciplinary approaches were emphasized, focusing on physical rehabilitation, cognitive therapy, mental health support, and symptom-targeted pharmacologic interventions. These strategies, detailed in studies following the 2003 SARS outbreak by Lam et al^19^ and Moldofsky and Patcai,^20^ laid the groundwork for understanding the complex needs of patients recovering from viral illnesses. However, emerging evidence suggests that long COVID exhibits a broader and more persistent symptom spectrum compared to post-SARS, affecting multiple organ systems and showing greater heterogeneity in clinical presentation.^21^ Long COVID necessitates more nuanced diagnostic tools and integrated, multidisciplinary care pathways that address its multifaceted manifestations.

Given that the pathophysiology of long COVID and its impact on sleep is not fully understood, the development of targeted treatments remains uncertain. Currently, there is no standardized protocol specifically addressing long COVID–associated sleep disturbances, though interventions involving nonpharmacological approaches are increasingly explored due to their generally lower risks and side effects.^22^ Guezguez et al^17^ suggest that recommendations endorsed by the European Academy for managing sleep problems during the pandemic can be extended to patients with long COVID. This includes cognitive behavioral therapy for insomnia, sleep hygiene improvement, and relaxation therapies. On the pharmacological side, National Center for Infectious Diseases physicians manage symptoms through the use of melatonin^7^ and antidepressants.^23^ However, the American Academy of Sleep Medicine recommends against the use of melatonin and trazodone for the treatment of chronic insomnia, citing insufficient evidence of efficacy.^24^ The application of these agents in long COVID may reflect off-label use driven by clinical need, although further research is warranted. This divergence reflects a broader lack of consensus on managing of long COVID–associated sleep disturbances, underscoring the need for high-quality studies to inform standardized care and determine the long-term effectiveness and safety of various interventions for sleep disturbances in long COVID.

This research aims to compile and evaluate current evidence on the interventions for sleep disturbances in patients with long COVID, through a systematic review and meta-analysis of randomized controlled trials. By assessing effectiveness, it seeks to identify promising interventions and pinpoint areas requiring further research, aiming to enhance clinical decision-making and care quality for affected individuals.

## METHODS

### Literature search and study selection

Two reviewers (D.Y.G. and W.C.L.) developed a comprehensive search strategy using a combination of relevant keywords (detailed in **Table S5** in the supplemental material). One reviewer (D.Y.G.) conducted a literature search across 6 databases including Embase, Web of Science, PubMed, Cochrane Library, China National Knowledge Infrastructure, and Wanfang Data from inception to December 2023.

Two reviewers (D.Y.G. and W.C.L.) independently screened the studies based on title and abstract, adhering to the inclusion and exclusion criteria. The studies included (1) were randomized control trials; (2) involved patients with confirmed COVID-19 infection; (3) included patients experiencing persistent symptoms, including sleep disturbance, for at least 4 weeks after initial SARS-CoV-2 infection (according to Centers for Disease Control and Prevention definition); (4) encompassed participants of both sexes; (5) encompassed participants ≥ 18 years of age; and (6) included interventions specifically addressing sleep disturbances associated with long COVID. Studies were excluded if they (1) were ongoing or incomplete; (2) presented as conference abstracts; (3) in the form of protocols; and (4) lacked a standardized scale for assessing sleep disturbances. No language restrictions were applied. Ongoing studies were reviewed but not included in the analysis. In cases of discrepancies in study inclusion, a third reviewer (L.L.D.Z.) provided adjudication.

### Data extraction

Selected studies underwent a full-text review to identify those eligible for data extraction. One reviewer (D.Y.G.) extracted relevant data from the included studies into a structured form, focusing on aspects such as study origin, inclusion and exclusion criteria, diagnostic protocols employed, participant demographics (age, sex, sample size, hospitalization status), intervention details (materials, duration), and outcomes. Data were categorized by intervention group and control group, with quantitative data presented as exact numbers reported or means with standard deviations and qualitative data were summarized. In cases of uncertainty, a second reviewer (W.C.L.) was consulted.

### Data analysis

The primary aim was to assess the effectiveness of interventions in addressing sleep disturbances among patients with long COVID, analyzing changes in standardized sleep scores reported in the studies. The secondary objective was to evaluate the safety of the interventions through the incidence of adverse events and serious adverse events, presented in a tabulated format for ease of interpretation.

Postintervention sleep scores for experimental and control groups were derived as mean values with standard deviations from each study. Per-protocol data were used, considering that most studies only included final data from participants who strictly adhered to intervention protocols. Meta-analysis was conducted on studies using the same standardized sleep scale and comparable interventions and controls. Mean differences along with corresponding 95% confidence intervals were calculated. A random-effects model was employed to address heterogeneity among studies, assessed using the inconsistency test (I^2^) with values more than 25% and 50% indicative of moderate and high heterogeneity, respectively. Forest plots were generated using the Cochrane Revman Web to visually represent effect size, 95% confidence intervals, and heterogeneity. Statistical significance was set at a *P* value below .05 for the overall effect.

Given there were fewer than 10 studies in the meta-analysis, assessment for publication bias did not apply to this study. This follows the guidelines outlined in the Cochrane Training Handbook.^25^

### Risk of bias assessment

One reviewer (D.Y.G.) evaluated the internal validity of the included studies using the Cochrane Risk of Bias Tool for randomized trials, version 2.0 (RoB 2.0). Risk of bias (RoB) across 5 domains, (1) randomization process, (2) deviations from intended interventions, (3) missing outcome data, (4) measurement of the outcome, and (5) selection of the reported result, was assessed using specific signaling questions. Each study’s influence in the analysis was weighted by its sample size. The assessment focused on the “per-protocol” effect, examining outcomes for participants who strictly adhered to the intervention. An overall RoB judgment (low, some concerns, or high RoB) was assigned to each study based on the domain-specific judgements. In cases of uncertainty, a second reviewer (W.C.L.) was consulted.

### Evaluation of literature quality

The GRADE (Grading of Recommendations, Assessment, Development, and Evaluation) system was used to evaluate the quality of evidence from the included studies. Each study was assessed based on 5 factors: (1) RoB, (2) consistency of results, (3) directness of results, (4) precision of estimates, and (5) publication bias.^22^ Each study was categorized as of low, moderate, or high quality.^26^ RoB was assessed using overall assessment from the RoB assessment (Section 2.4), where studies with green ratings indicated low risk, yellow suggested some concerns, and red indicated high risk. Consistency was evaluated based on variability across studies, directness was assessed by study relevance to sleep disturbances in patients with long COVID, and precision was determined by sample sizes and confidence intervals. Finally, publication bias was assessed by checking for selective reporting of results. This system aided in assessing the reliability of interventions.

## RESULTS

### Literature search results

**Figure 1:**
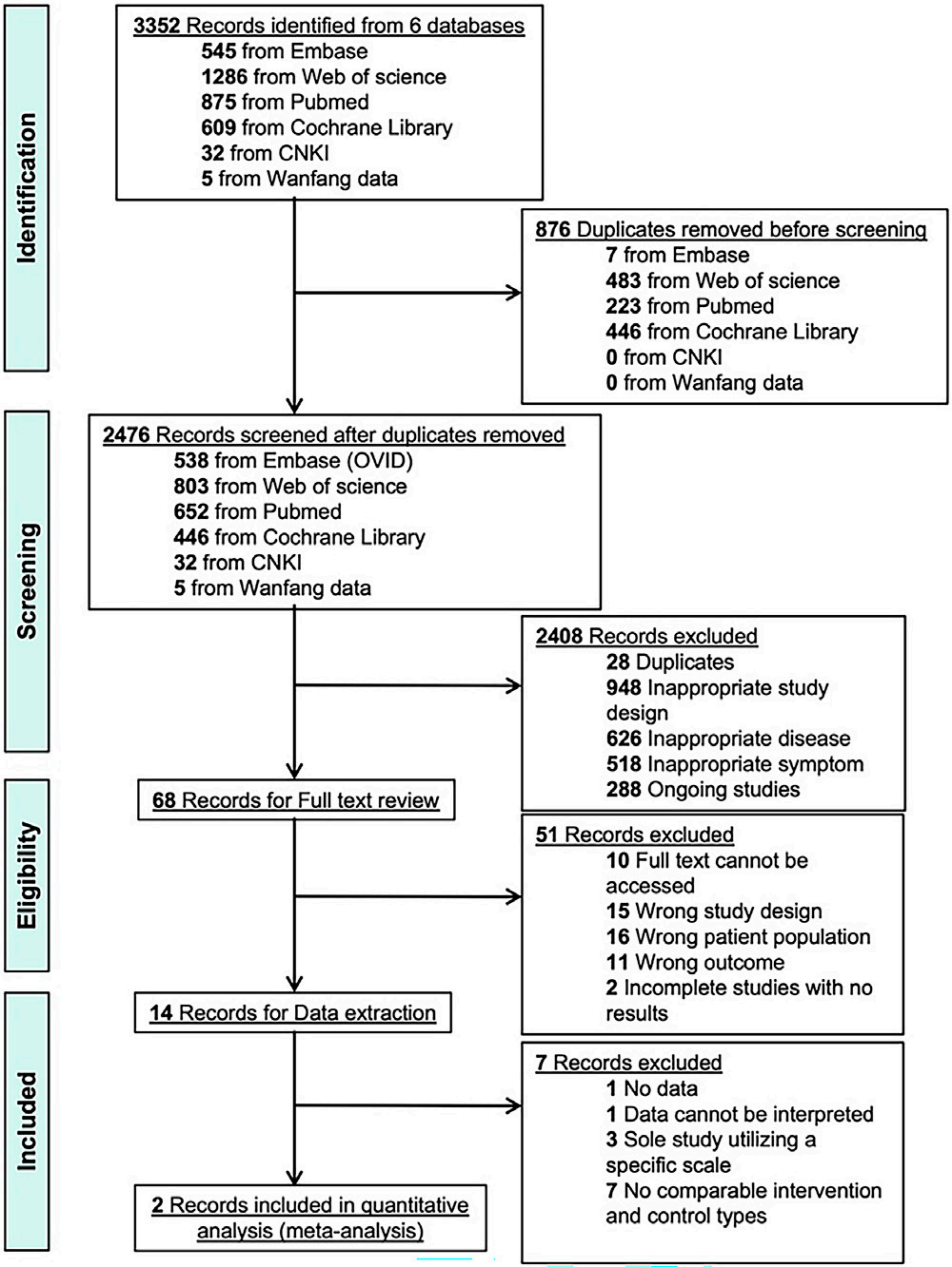
Flow diagram for study selection and inclusion.

**Figure 1** outlines the search process for relevant studies. A total of 3,352 studies were initially identified from the systematic database search, with 876 duplicates removed, leaving 2,476 studies for title and abstract screening. After excluding 28 additional duplicates and 2,380 irrelevant studies, 68 studies underwent full-text review. Of these, 14 studies met our inclusion criteria for systematic review, and only 2 were eligible for meta-analysis. The primary reasons for exclusion were studies not involving long COVID (n = 16) and failure to adhere to randomized controlled trial (RCT) design criteria (n = 15).

### Characteristics of included studies

**Table 1** summarizes the main characteristics of the 14 included studies, involving 1,975 patients with long COVID. Sleep disturbances, predominantly poor sleep quality and insomnia, were measured using standardized sleep scales including the Pittsburgh Sleep Quality Index (PSQI) in 7 studies, the Insomnia Sleep Index (ISI) in 2 studies, the Visual Analog Scale (VAS) in 2 studies, and other questionnaires (Spiegel Sleep Quality Questionnaire, Postacute COVID-19 Syndrome Questionnaire [PACSQ-14], and the Korean Version of Modified Leeds Sleep Evaluation Questionnaire [KMLSEQ]) in 3 studies.

**Table 1:** Summary of included studies.

Study	Country	Sample Size	Age (mean ± SD, year)	Females (%)	Hospitalization Status	IG Intervention	CG Intervention	Treatment Duration (days)	Outcome	Assessment Scale	Cut-Off for Poor Sleep	MCID for Clinically Meaningful Treatment Response
Tanashyan et al 2022	Russia	159 IG: 80 CG: 79	IG: 44.5 CG: 44.5	IG: 73.75 CG: 77.21	NR	Brainmax	Placebo	< 55	Sleep quality	PSQI	≥ 5	−4.4
Putilina et al 2021	Russia	100 IG: 50 CG: 50	IG: 40.4 ± 11.7 CG: 40.4 ± 13.3	IG: 49 CG: 37	Outpatients - 17 in experimental group, 9 in control group	Cytoflavin tablets	Other medications (vitamins, nootropic drugs)	25	Sleep quality	PSQI	≥ 5	−4.4
Hajibashi et al 2023	Iran	52 IG: 26 CG:26	IG: 44.19 ± 8.40 CG: 46.00 ± 11.26	IG: 50.0 CG: 42.3	Discharged	Pulmonary telerehabilitation & progressive muscle relaxation	Pulmonary telerehabilitation	30	Sleep quality	PSQI	≥ 5	−4.4
Keskin and Saka, 2023	Turkey	76 IG: 38 CG: 38	IG: 38.65 ± 11.56 CG: 36.36 ± 10.97	IG: 39.47 CG: 39.47	Outpatient	Strengthening exercises & relaxation exercises	No exercise program	24	Sleep quality	PSQI	≥ 5	−4.4
Tartibian et al 2022	Iran	261 IG: moderate-intensity continuous training (MICT) (60); resistance training (RT) (61); combined aerobic and resistance training (CET) (72) CG: 65	IG: MICT (55.0 ± 29.1); RT (56.2 ± 26.9); CET (59.6 ± 34.3) CG: 59.4 ± 36.5	IG: MICT (28.57); RT (19.67); CET (23.61) CG: 29.23	Discharged	Home-based MICT Home-based RT Home-based CET	Nonexercise (NON-EX)	24	Sleep quality	PSQI	≥ 5	−4.4
Zilberman-Itskovich et al 2022	Israel	73 IG: 37 CG: 36	IG: 48.4 ± 10.6 CG: 47.8 ± 8.5	IG: 51.4 CG: 69.4	Hospitalized (4 in experimental group, 3 in control group)	Hyperbaric oxygen therapy	Sham (breathing 21% oxygen)	40	Sleep quality	PSQI	≥ 5	−4.4
Mosavi et al 2023	Iran	60 IG: MBSR (15); Aerobic exercise (15); Combination (15) CG: 15	IG: NR CG: NR	IG: NR CG: NR	Discharged	Mindfulness-based stress reduction (MBSR) Aerobic exercise Combination of MBSR and aerobic exercise	Control group	14	Sleep quality	PSQI	≥ 5	−4.4
An et al 2022	China	196 IG: 99 CG: 97	IG: 54.5 ± 10.8 CG: 55.0 ± 9.4	IG: 73.7 CG: 67.0	NR	Shugan Jieyu capsule	Placebo	41	Insomnia	ISI	> 7	−6
Deshpande et al 2022	India	74 IG: 37 CG: 37	IG: 40.54 ± 12.05 CG: 37.24 ± 9.57	IG: 32.43 CG: 48.65	Hospitalized	Immunodaat capsules & conventional management for COVID-19	Conventional COVID-19 management (vitamin B complex, vitamin C, zinc supplements/ medications as per symptom requirement)	30	Insomnia	ISI	> 7	−6
Li et al 2021	China	182 IG: 96 CG: 86	IG: 55 CG: 55	IG: 57.14 CG: 65.66	Discharged	Shumian capsule	Shumian capsule simulator	14	TCM symptom of insomnia	VAS	≥ 4	N.A.
Yang et al 2023	China	185 IG: 96 CG: 89	IG: 54 CG: 54	IG: 65.00 CG: 54.00	Discharged	Xiaoyao capsule	Placebo	14	Self-assessed sleep quality	VAS	≥ 4	N.A.
Hausswirth et al 2023	France	34 IG: 17 CG: 17	IG: 47.1 ± 8.3 CG: 48.7 ± 10.4	IG: 76.47 CG: 70.59	Hospitalized	Neuro-mediation program	No treatment	10	Sleep quality	Spiegel Sleep Quality questionnaire (SSQ)	≤ 17	N.A.
Lau et al 2023	Hong Kong	463 IG: 232 CG: 231	IG: 49.3 CG: 49.6	IG: 66 CG: 65	Hospitalized	SIM01	Placebo (vitamin C)	189	Insomnia	PACSQ-14 questionnaire	N.A.	N.A.
Kang et al 2023	Korea	60 IG: 30 CG: 30	IG: 31.30 ± 11.03 CG: 31.63 ± 8.67	IG: 73 CG: 70	Discharged	Aromatherapy	Usual care	4	Sleep quality	Korean Version of Modified Leeds Sleep Evaluation Questionnaire (KMLSEQ)	N.A.	N.A.

CG = control group, IG = intervention group, ISI = Insomnia Sleep Index, MCID = minimum clinically important difference, NR = no report, PACSQ-14 = Patient’s Assessment of Sleep Quality (14-item), PSQI = Pittsburgh Sleep Quality Index, SD = standard deviation, TCM = Traditional Chinese Medicine, VAS = Visual Analog Scale, N.A. = Not Applicable.

To enhance the interpretability of treatment outcomes, established thresholds for clinical significance were applied where available. For the PSQI, a global score greater than 5 is widely recognized as the clinical cutoff for distinguishing “poor” from “good” sleepers. This threshold has been validated across diverse populations as supported by Curcio et al,^27^ who demonstrated high diagnostic sensitivity and specificity for identifying significant sleep disturbances. Additionally, a reduction of approximately 4.4 points on the PSQI is commonly cited as the minimum clinically important difference (MCID), the smallest change perceived as beneficial by patients.^28^ This value is particularly relevant for assessing whether an intervention yields meaningful improvement in sleep quality. For the ISI, a score above 7 indicates clinically significant insomnia. A reduction of 6 points or more on the ISI is generally considered the MCID, signifying a substantial and clinically meaningful treatment response.^29^ On the other hand, the VAS, Spiegel Sleep Quality (SSQ), PACSQ-14, and KMLSEQ scales lack standardized MCID or clinical cutoff values, which limits their utility for interpreting treatment effects in a clinically meaningful way. In this review, we prioritized the use of MCID values to evaluate the clinical relevance of intervention effects. Outcomes exceeding these thresholds were interpreted as reflecting meaningful improvements from the patient’s perspective.

Patient status varied across studies, with 6 studies including discharged patients, 4 with hospitalized patients, 2 with outpatients, and 2 not reporting hospitalization status. Interventions for long COVID–associated sleep disturbances ranged from 4–189 days. Pharmacological interventions targeting cellular oxygen metabolism were used in 2 studies, and nonpharmacological interventions in the other 12 studies included natural remedies in 7 studies, therapeutic exercise in 3 studies, aerobic exercise with lifestyle modification in 1 study, and stimulation therapy with mindfulness training in 1 study.

### Effectiveness of pharmacological interventions

**Table S1** in the supplemental material presents the effectiveness of the pharmacological interventions in improving the outcome of sleep quality in patients with long COVID. These interventions (detailed in **Table S6** in the supplemental material) enhance cellular oxygen metabolism through distinct mechanisms. Treatment durations ranged from 25 to less than 55 days, involving 130 participants, with notable effectiveness observed in individuals in their 40s. It should be noted that the mechanistic claims described in **Table S1** and **Table S6** are based on information reported by the study authors and have not been evaluated or endorsed by regulatory authorities such as the Food and Drug Administration.

#### Sleep quality measured by PSQI

Two studies using the PSQI reported statistically significant improvements in sleep quality following intervention. In the study by Tanashyan et al,^30^ participants who received Brainmax showed a mean reduction in PSQI score of 2.5 points, compared to no change in the control group. The difference between the 2 groups was statistically significant (*P* < .001). However, this improvement did not meet the MCID threshold of 4.4 points, suggesting that the observed change, although statistically robust, may not represent a clinically meaningful improvement in sleep quality in the patients. Despite this, the greater reduction in the intervention group compared to control group indicates a positive treatment effect.

In contrast, Putilina et al^31^ evaluated the effects of Cytoflavin and reported a mean PSQI score reduction of 11.36 in the intervention group, compared to 5.12 in the control group. This difference was statistically significant (*P* < .05) and the reduction in the intervention group exceeded the MCID threshold of 4.4 more greatly, indicating a clinically meaningful improvement in sleep quality.

### Effectiveness of nonpharmacological interventions

**Table S2** in the supplemental material reveals that 10 out of 12 studies showed improvements in sleep quality and insomnia, demonstrating the overall effectiveness of nonpharmacological interventions for long COVID–associated sleep disturbances. Treatment durations varied from 4–189 days, employing diverse methods that enhanced physical fitness, reduced stress and anxiety, boosted immune function, and modified neurochemical pathways (detailed in **Table S7** in the supplemental material). These interventions demonstrated broad effectiveness across 15 to 232 participants, notably in individuals aged 30 to 59 years. It is to note that the mechanistic claims described in **Table S2** and **Table S7** are based on information reported by the study authors and have not been evaluated or endorsed by regulatory authorities such as the Food and Drug Administration.

#### Sleep quality measured by PSQI

Five studies evaluated sleep quality using the PSQI, with all reporting significant improvements. Three studies focused on therapeutic exercise.

Hajibashi et al^32^ investigated the combined effects of Progressive Muscle Relaxation (PMR) and pulmonary telerehabilitation (PTR). PMR was also associated with reductions in anxiety, depression, and fatigue, and PTR improved functional capacity and reduced anxiety symptoms. The intervention group receiving both PMR and PTR reported a mean PSQI score reduction of 3.08, compared to 1.16 in the control group receiving PTR alone. Although this improvement was statistically significant (*P* = .001), it did not reach the MCID threshold of 4.4, indicating that the combined intervention, although more effective than PTR alone, may not have provided a clinically meaningful improvement in sleep quality.

Keskin^33^ demonstrated the positive impact of physical activity combined with chronobiotic light exposure on pain and sleep quality. Participants in the intervention group who engaged in strengthening and relaxation exercises experienced a mean PSQI score reduction of 2.89, compared to 0.37 in the control group. This difference was statistically significant (*P* < .001) but fell short of the MCID threshold, suggesting a positive effect of exercise, though not large enough to be considered clinically meaningful.

Tartibian et al^34^ evaluated different exercise modalities, including moderate-intensity continuous training, resistance training, and concurrent exercise training, over 20–30 days. Although specific PSQI scores were not reported, concurrent exercise training was noted to have the most pronounced impact on sleep quality. The authors suggested that the combination of aerobic and resistance training may be particularly effective in improving sleep, likely through its benefits on immune function, inflammation, and cardiovascular and renal health.

Zilberman-Itskovich et al^35^ examined hyperbaric oxygen therapy administered over 40 days as a natural intervention. The study proposed that hyperbaric oxygen therapy may enhance sleep through improved brain perfusion and microstructural changes. Patients in the treatment group reported a mean PSQI score reduction of 2.6, compared to 1.0 in the sham control group. The difference was not statistically significant (*P* = .704) and did not meet the MCID threshold, suggesting that although some improvement was noted, the evidence does not support hyperbaric oxygen therapy to provide clinically meaningful sleep improvements.

Mosavi et al^36^ investigated the effects of combining aerobic exercise with mindfulness practices over a 14-day period. Although mean PSQI reductions were not reported, participants who engaged in both interventions reported greater improvements in sleep quality and stress reduction compared to those who received either intervention alone. These findings suggest a synergistic benefit of combining physical and psychological therapies.

#### Sleep quality and the Traditional Chinese Medicine symptom of insomnia measured by VAS

Two studies used the VAS to measure the sleep quality and the Traditional Chinese Medicine (TCM) symptom of insomnia. Both studies employed natural remedies targeting liver function over 14 days.

Li et al^37^ evaluated the effects of the Shumian capsule on insomnia and mental health, which is formulated to soothe liver function, alleviate depression and anxiety, and calm the mind. Whereas both the intervention and control groups showed improvements in sleep quality as measured by the VAS, the mean score reduction in the intervention group was 3.1 points compared to 2.1 points in the control group, with the difference reaching statistical significance (*P* = .001). Despite this, the change did not exceed the MCID threshold of 4.4, suggesting that although the Shumian capsule may offer statistically meaningful improvements, its clinical benefit in treating insomnia may be limited.

In contrast, Yang et al^38^ investigated the Xiaoyao capsule, which targets liver stagnation and supports spleen, blood, and menstrual function. Over a 14-day period, the intervention group demonstrated a 3.4-point reduction in VAS scores, compared to a 3.3-point reduction in the control group. This minimal difference was not statistically significant (*P* > .05), suggesting that although both groups experienced symptomatic improvement the data do not support a clinically meaningful benefit attributable to the Xiaoyao capsule.

#### TCM symptom of insomnia and poor sleep measured by TCM syndrome scale

Li et al^37^ and Yang et al^38^ also used the TCM syndrome scale to assess insomnia and poor sleep symptoms over 14 days. Both studies found greater benefits in the intervention group compared to the control group, though differences were not statistically significant. Li et al^37^ observed that the therapeutic effects of the Shumian capsule became apparent only after 14 days, with 1 participant in the intervention group achieving complete resolution of insomnia. Similarly, Yang et al^38^ reported that the Xiaoyao capsule was associated with the resolution of poor sleep in one participant after 7 days, and in 3 participants in both intervention and control groups by day 14. These findings suggest that although both interventions may exert mild-to-moderate benefits over time, the evidence remains limited due to the small magnitude of change and lack of statistical significance.

#### Sleep quality measured by SSQ

Hausswirth et al^39^ employed the SSQ questionnaire to evaluate sleep quality addressed by the Neuro-Mediation Program. This intervention combines noninvasive cognitive stimulation and mindfulness training with sound and light therapy over 10 days to treat long COVID symptoms. The study reported statistically significant improvements in sleep quality (*P* = .089), alongside reductions in physical and mental fatigue, muscle and joint pain, mood disturbances, depression and anxiety. The mean SSQ score increased from approximately 15.1 to 18.5 in the intervention group, compared to a smaller increase from 14.0 to 16.0 in the control group. This greater improvement in the intervention group, which also surpassed the cut-off score of 17 for poor sleep, indicates enhanced sleep quality experienced by participants who underwent the Neuro-Mediation Program.

#### Sleep quality measured by KMLSEQ

Kang et al^40^ assessed sleep quality using the KMLSEQ. The study examined aromatherapy, specifically inhalation of an essential oil blend of lavender and ylang-ylang in an 8:2 ratio, over 4 days. Lavender, rich in linalyl acetate, is known for its calming effects on the nervous system, and ylang-ylang, which contains benzyl acetate, has recognized soothing properties. Although participants in the aromatherapy group reported greater improvements in sleep quality compared to the control group, the difference did not reach statistical significance (*P* = .089), indicating that the intervention may not produce a reliably measurable benefit in sleep outcomes.

#### Insomnia measured by ISI

Two studies by An et al^41^ and Deshpande et al^42^ assessed interventions using natural ingredients for insomnia with the ISI. Both reported statistically significant improvements in sleep quality.

An et al^41^ studied the use of Shugan Jieyu capsules, formulated to regulate the sleep cycle, over 41 days. The intervention group demonstrated a mean ISI score reduction of 11.2 points compared to 2.6 points in the control group, a statistically significant difference (*P* < .001). This reduction exceeded the MCID of 6 points, indicating a clinically meaningful treatment response.

Deshpande et al^42^ examined the effects of Immunodaat capsules, which contain elderberry extract, administered over a 30-day period. Elderberry’s phytonutrients, vitamins, and anthocyanins are known to support immune function, reduce inflammation, and alleviate symptoms such as pain and fever. The study reported a mean ISI score reduction of 1.29 points in the intervention group vs 0.75 points in the control group, with a statistically significant difference (*P* < .05). However, this change did not reach the MCID threshold, suggesting that although the intervention produced a measurable effect it may not represent a clinically meaningful improvement in insomnia.

#### Insomnia measured by PACSQ-14

Lau et al^43^ used the PACSQ-14 to measure SIM01’s effect on insomnia. Over 189 days, SIM01 was associated with improvements in sleep in both the intervention and control groups. Participants in the intervention group reported a 58% reduction in insomnia symptoms, significantly greater than the 44% reported in the control group (*P* < .05).

### Meta-analysis

**Figure 2:**
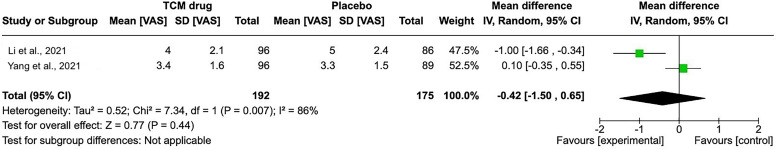
Forest plot of sleep quality measured by VAS. CI = confidence interval, SD = standard deviation, VAS = Visual Analog Scale.

The meta-analysis (**Figure 2**) assessed the effectiveness of TCM drugs vs placebo for managing long COVID–associated sleep disturbances using VAS scores. The analysis included 192 patients receiving the TCM drugs and 175 individuals receiving placebo. In Li et al^37^ the TCM drug group had a lower mean VAS score compared to placebo, but this was not seen in Yang et al.^38^ The pooled mean difference of −0.42 (95% confidence interval: −1.50, 0.65) was not statistically significant (*P* = .44) and showed substantial heterogeneity (I^2^ = 86%, *P* = .007), suggesting that the observed differences between 2 groups in both studies could be due to chance and that effectiveness of the TCM drugs may not be supported. Additionally, subgroup analysis by intervention type was not applicable as both studies involved pharmacological interventions.

### Adverse events and serious adverse events analysis

The evaluated interventions across the 14 studies generally exhibited a favorable safety profile (**Table S3** in the supplemental material). Adverse events were reported in 6 studies, though none required discontinuation of the interventions or showed statistically significant differences between the treatment and control groups. No serious adverse events were observed. For instance, Zilberman-Itskovich et al^28^ reported barotrauma and allergic reactions from hyperbaric oxygen therapy, but these were not significantly different from the control group, and no patients discontinued treatment due to side effects, suggesting manageable safety concerns. In studies by An et al^41^ and Kang et al^40^ the reported minor adverse events that did not necessitate discontinuation of the intervention. Furthermore, 4 studies reported no adverse events or serious adverse events, and the remaining 4 studies did not report any safety events.

### RoB assessment

**Figure 3:**
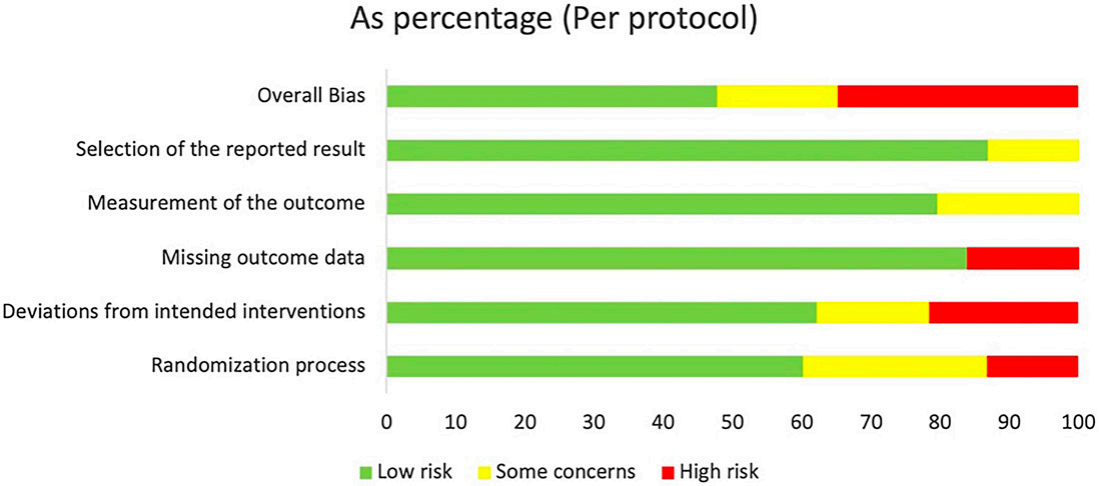
Percentage distribution of studies across varying risk levels.

**Figure 3** presents the overall RoB assessment of the included studies, indicating a predominantly low RoB, with 40–50% of studies having low overall bias. Domains such as “Selection of the reported result” and “Measurement of the outcome” indicated minimal selection bias and reliable outcome measurement, thus enhancing the validity of the reported results. The “Missing outcome data” domain generally shows reliable handling of missing data, though some issues are noted. Conversely, the “Deviations from intended interventions” domain presents the highest RoB, suggesting some studies experienced deviations that affected outcomes. The “Randomization process” domain shows a higher risk comparatively, indicating risks in the randomization process for some studies.

**Figure 4:**
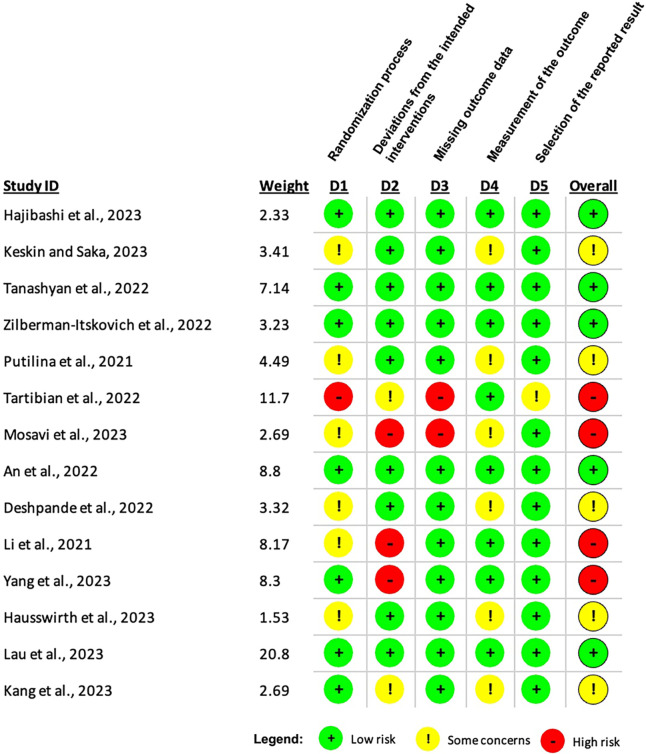
Tabulated summary of the risk of bias for individual studies.

**Figure 4** presents the RoB assessment for each study. Five studies were deemed to have a low risk, 5 had some concerns, and 4 had high risk. Notable flaws were identified in the “Deviations from the intended interventions” and “Missing outcome data” domains, with 3 and 2 high-risk studies, respectively. For example, Tartibian et al^34^ and Mosavi et al^36^ had high risks due to poor handling of missing data, issues in the randomization process, and deviations from the intended interventions. Contrarily, studies like Tanashyan et al^30^ and Hajibashi et al,^32^ which had low RoB across all domains, were well-conducted and provided robust and reliable evidence.

### GRADE assessment

The GRADE assessment (**Table S4** in the supplemental material) reveals that 5 studies were of moderate quality and 9 were of low quality. The difference in quality stemmed from the RoB. Studies of moderate quality demonstrated low RoB due to thorough randomization and robust data handling, whereas studies of low quality had high RoB due to significant concerns in multiple domains. Inconsistency was “not serious” across all studies because results did not vary significantly. All studies directly addressed sleep disturbances in patients with long COVID using interventions, showing no serious indirectness. Imprecision was a concern for all studies, varying from more to less serious, due to small sample sizes and wide confidence intervals. Publication bias was “undetected” for all studies because there was no evidence of selective reporting or nonpublication of negative findings.

## DISCUSSION

### Summary of findings

This study represents a pioneering effort to systematically assess and synthesize existing evidence on interventions aimed at mitigating sleep disturbances in patients with long COVID. Through a comprehensive search strategy across multiple databases, we identified and evaluated relevant RCTs. Our comparative analysis of the studies highlights considerable variations in treatment effectiveness and duration.

#### Pharmacological interventions

The review found some support for Brainmax and Cytoflavin in improving sleep quality by enhancing cellular oxygen metabolism. When comparing the 2 interventions, Tanashyan et al’s^30^ study on Brainmax was deemed more reliable, with a low RoB and moderate quality evidence. In contrast, Putilina et al’s^31^ study on Cytoflavin reported both clinically and statistically meaningful improvements in sleep quality. Brainmax is thought to improve sleep by enhancing mitochondrial respiration and cellular function under stress, attributed to its succinic acid complex and trimethylhydrazinium components that support anaerobic metabolism and energy exchange during hypoxia. This addresses the physiological disruptions caused by long COVID that contribute to sleep disturbances. In a similar manner, Cytoflavin is believed to improve cellular energy production, reduce oxidative stress, and support cognitive function by restoring antioxidant defenses and enhancing glucose and fatty acid metabolism. Both interventions show promise as potential treatments for long COVID–related sleep disturbances; however, further high-quality studies are needed to confirm their efficacy and long-term safety.

#### Nonpharmacological interventions

The review supports the effectiveness of nonpharmacological interventions, with 10 out of 12 studies showing improvements in sleep quality or insomnia.

Three studies highlighted the effectiveness of therapeutic exercises involving aerobic, resistance, strength exercises, and muscle relaxation. Among these studies, Hajibashi et al^32^ provided higher-quality evidence with a low RoB, identifying PTR and PMR as promising treatments for long COVID–associated sleep disturbances. PTR combines breathing exercises and strength training to enhance functional capacity, improve quality of life, and alleviate anxiety. PMR involves muscle contraction and relaxation exercises, reducing anxiety and improving sleep quality by addressing the psychological stress linked to long COVID–associated sleep disturbances. Although the combination of PTR and PMR did not yield a clinically significant improvement in sleep outcomes, both interventions show potential for supporting sleep and overall recovery by addressing the physical and psychological challenges commonly experienced by patients with long COVID, particularly those with reduced fitness and elevated anxiety levels.

Seven studies explored natural remedies. Among these, studies by Zilberman-Itskovich et al^35^ and Lau et al^43^ had low RoB and moderate-quality evidence. Nevertheless, data in Zilberman-Itskovich et al^35^ did not support hyperbaric oxygen therapy as an effective therapy for clinically meaningful sleep improvements in patients with long COVID. On the other hand, although the PACSQ-14 scale used in Lau et al^43^ lacks a validated MCID, the study reported greater alleviation of patients with insomnia treated with SIM01. SIM01, a synbiotic preparation containing *Bifidobacterium* strains and prebiotics, targets gut dysbiosis to modify the immune response and alleviate long COVID symptoms. SIM01 provided additional health benefits, including enhanced gut microbiota diversity, an increase in short-chain fatty acid–producing bacteria, and a decrease in antimicrobial resistance gene prevalence. The prebiotic components further supported the growth of beneficial bacteria while inhibiting pathogenic species.

An et al’s^41^ study on the Shugan Jieyu capsule demonstrated strong findings, supported by a low RoB and moderate-quality evidence. This traditional formulation, intended to enhance liver function and exert antidepressant effects, includes key herbal components such as Guan ye lian qiao and Ciwujia. These ingredients are believed to support the liver, spleen, kidney, and heart, while also exhibiting sedative properties and contributing to sleep regulation. The study reported both statistically and clinically significant improvements in sleep-related outcomes, highlighting the capsule’s potential as a therapeutic option for managing long COVID–associated sleep disturbances.

In contrast, two studies by Kang et al^40^ and Yang et al^38^ on aromatherapy and the Xiaoyao capsule were ineffective. Kang et al^40^ attributed the nonsignificant effect of aromatherapy to a small patient population, and the Xiaoyao capsule’s lack of improvement could be due to its slow absorption over the 2-week treatment period. The ineffectiveness of the Xiaoyao capsule is further supported by the meta-analysis. The meta-analysis of revealed heterogeneity in the effects of Shumian and Xiaoyao capsules, providing no conclusive evidence of their effectiveness. The complexity of TCM formulas pose challenges for scientific studies, as they contain numerous herbal ingredients with varying bioactive compounds and inconsistent chemical compositions.^44^

Two studies by Tartibian et al^34^ and Mosavi et al^36^ on aerobic exercise combined with mindfulness training and stimulation therapy with mindfulness training showed sleep improvements but were of low quality with a high RoB, thus not strongly supported as potential treatments.

#### Assessment of outcomes

Different measurement tools yielded varied results in evaluating the outcomes of long COVID–associated sleep disturbances. The PSQI was used in 50% of the studies (7 out of 14), consistently showing significant sleep improvements in both low and moderate RoB studies. Twelve out of the 14 studies reported only 1 sleep outcome using a single scale, whereas Li et al^37^ and Yang et al^38^ offered more comprehensive evaluations by using both the VAS and TCM symptom scale. This underscores the need for more standardized tools to accurately assess the effectiveness of interventions for sleep disturbances in patients with long COVID.

Overall, 12 studies reported sleep disturbances alongside other long COVID symptoms. In particular, anxiety was noted in 10 studies, measured by various scales including the VAS (3 studies), Beck Anxiety Inventory (2 studies), Hospital Anxiety and Depression Scale (2 studies), TCM symptom scale (2 studies), Brief Symptom Inventory (1 study), Quality of Life questionnaire (1 study), and Hamilton Anxiety Scale (1 study). Interventions significantly reduced anxiety in 9 studies, suggesting that improvements in sleep disturbances may be linked to effective anxiety management in patients with long COVID.

### Ongoing studies

The assessment of ongoing studies illustrates the exploration of additional treatments for long COVID–associated sleep disturbances. A phase 2 RCT in Hong Kong is comparing individualized TCM treatments to conventional Western medicine over 4 weeks, using the Modified COVID-19 Yorkshire Rehabilitation Scale (C19-YRSm) to assess impact on sleep quality and overall symptom management.^45^ In the United Kingdom, a phase 3 trial is investigating Ashwagandha, a traditional Indian herb, in 2,500 adults with long COVID. Self-reported sleep quality will be measured using the Patient-Reported Outcomes Measurement Information System 29 + 2 individual dimensions over 3 months.^46^ In the United States, a phase 3 feasibility trial is testing histamine receptor antagonists, cetirizine and famotidine, in survivors of COVID-19 with sleep disturbances over 12 weeks. Sleep outcomes will be measured using the ISI.^47^ These studies showcase diverse approaches, from traditional and herbal medicines to pharmacological treatments, in addressing long COVID–associated sleep disturbances.

### Strengths and weaknesses

The study adopts a comprehensive approach with an extensive search across six English and Chinese databases to ensure wide coverage of the available literature on long COVID–associated sleep disturbances. Conducting a meta-analysis enhances statistical power through quantitative synthesis of data from individual RCTs, proving precise estimates of treatment effects. Additionally, the included studies were selected based on uniform symptom terminology, standardized recording methods, and consistent grouping criteria.

Despite the positive findings, this review acknowledges several limitations. First, the dataset comprised fewer than 20 RCTs, with only 14 RCTs included in the systematic review, which potentially affects the robustness of the conclusions in this study. Moreover, meta-analysis of only 2 studies suggests insufficient evidence of the effectiveness of the TCM drugs. This limited pool reflects challenges in conducting trials during the COVID-19 pandemic period and the nascent and developing nature of research in this field. The small number of studies underscores the urgent need for more high-quality, large-scale trials to address long COVID–related sleep disturbances. Nonetheless, the current analysis offers a critical synthesis of early evidence and sets a foundation for future research.

Second, the inclusion of studies with varied quality reveals significant methodological concerns, contributing to imprecision of the results. High risks of bias, particularly from “Deviations from the intended interventions” and “Missing outcome data” domains, suggest problems with adherence to interventions and data handling that affected reliability of the findings. Furthermore, relying on per-protocol analysis compromises the internal validity of the findings, because it reduces the benefits of randomization and may bias treatment effects.

Third, limitations exist in the sleep assessment tools used for long COVID. Although the PSQI is commonly applied, its broad scope may not fully capture insomnia-specific symptoms relevant to long COVID. The ISI, by contrast, offers greater specificity. Future studies should consider analyzing PSQI subcomponents or incorporating more targeted or complementary tools. Additionally, other scales such as the VAS, SSQ, KMLSEQ, and TCM-based symptom scales lack validated MCIDs, limiting interpretability and comparability. Establishing standardized, clinically meaningful thresholds is essential for improving the assessment of sleep disturbances in long COVID.

### Future research

Further research could prioritize larger sample sizes, with over 10 studies in the meta-analysis for a publication bias assessment to provide more conclusive evidence of the interventions’ effectiveness.^25^ Moreover, variation in treatment durations across the included studies underscores the need to examine duration-dependent effectiveness in larger trials. Whereas current research focuses predominantly on nonpharmacological approaches, future studies should also explore more conventional pharmacological treatments for long COVID–associated sleep disturbances. This would provide a more balanced understanding of potential interventions.

To improve intervention adherence, future studies could conduct regular follow-ups using tools such as wearable devices for monitoring. Implementing rigorous randomization and blinding procedures, alongside detailed protocols will reduce biases and improve the consistency of the results. To better handle missing data, transparent reporting and employing statistical methods such as multiple imputation and sensitivity analyses will further strengthen the reliability of findings.^48^^,^^49^ Moreover, flexible treatment regimens can be done to reduce the occurrence of and mitigate the impact of missing data.

## CONCLUSIONS

In conclusion, this systematic review and meta-analysis provide insights into the available interventions for managing sleep disturbances in patients with long COVID. Both pharmacological and nonpharmacological interventions demonstrate varying effectiveness, with an emphasis on nonpharmacological approaches. Although definitive evidence was limited due to sample size constraints, potential treatments identified set the direction for future research. As treatment strategies for long COVID evolve, these findings enhance the understanding of the condition and improve patient outcomes.

## DISCLOSURE STATEMENT

All authors have seen and approved the manuscript. This research was supported by Nanyang Technological University under the URECA Undergraduate Research Programme, the Chinese Medicine Development Fund (Ref. No. 22B2-011A), and the Health and Medical Research Fund (Ref. No. 20211471). The authors report no conflicts of interest.

## OPEN ACCESS

Copyright 2025 The Authors. This is an open access article, distributed under the Creative Commons Attribution 4.0 International License. Sharing and adaptation are permitted provided attribution to its original publication in the Journal of Clinical Sleep Medicine is made in accordance with the license.

## Supplemental Materials

10.5664/jcsm.11782 Supplemental Materials

## ABBREVIATIONS

GRADE: Grading of Recommendations, Assessment, Development, and Evaluation
ISI: Insomnia Sleep Index
KMLSEQ: Korean Version of Modified Leeds Sleep Evaluation questionnaire
MCID: minimum clinically important difference
PACSQ-14: Patient’s Assessment of Sleep Quality (14-item)
PMR: progressive muscle relaxation
PSQI: Pittsburgh Sleep Quality Index
PTR: pulmonary telerehabilitation
RCT: randomized controlled trial
RoB: risk of bias
SSQ: Spiegel Sleep Quality questionnaire
TCM: Traditional Chinese Medicine
VAS: Visual Analog Scale
