# Supplementary material for: Effect of interventions for the management of sleep disturbances in patients with long COVID: a systematic review and meta-analysis of randomized controlled trials
Source: J Clin Sleep Med. 2025 Nov 1;21(11):1993–2005. doi: 10.5664/jcsm.11782 (PMC12582205; doi:10.5664/jcsm.11782)
Supplement: Supplementary file 1 [file jcsm.11782.sm001.pdf]

Table S1

Study	IG Intervention	IG Sample size	IG Age (mean ± SD)	Treatment duration (days)	Outcome	Assessment scale	Mean score difference reported for IG	Mean score difference reported for CG	p- value for difference between IG and CG	Effect of intervention
Tanashyan et al., 2022	Brainmax	80	44.5 ± 0.0	< 55	Sleep quality	PSQI	-2.5	-0	<0.001	Improved sleep<br><br>**(Target cellular oxygen metabolism)**
Putilina et al., 2021	Cytoflavin tablets	50	40.4 ± 11.7	25	Sleep quality	PSQI	-11.36	-5.12	<0.05	Improved sleep<br><br>**(Target cellular oxygen metabolism)**

Table S2

Study	IG Intervention	IG Sample size	IG Age (mean ± SD)	Treatment duration (days)	Outcome	Assessment scale	Mean score difference reported for IG	Mean score difference reported for CG	p- value for difference between IG and CG	Effect of intervention
Hajibashi et al., 2023	Pulmonary telerehabilitation & Progressive muscle relaxation	26	44.19 ± 8.40	30	Sleep quality	PSQI	-3.08	-1.16	0.001	Improved sleep<br><br>**(Therapeutic exercise)**
Keskin and Saka, 2023	Strengthening exercises & Relaxation exercises	38	38.65 ± 11.56	24	Sleep quality	PSQI	-2.89	- 0.37	< 0.001	Improved sleep<br><br>**(Therapeutic exercise)**
Tartibian et al., 2022	Home-based moderate-intensity continuous training (MICT)<br><br>Home-based Resistance training (RT)<br><br>Home-based Combined aerobic and resistance training (CET)	MICT - 63<br><br>RT - 61<br><br>CET - 72	MICT - 55.00 ± 29.10<br><br>RT - 56.20 ± 26.90<br><br>CET - 59.60 ± 34.30	24	Sleep quality	PSQI	NR	NR	> 0.05	CET improved sleep the most<br><br>**(Therapeutic exercise)**
Zilberman-Itskovich et al., 2022	Hyperbaric oxygen therapy	37	48.40 ± 10.60	40	Sleep quality	PSQI	-2.6	-1.0	0.704	Improved sleep<br><br>**(Natural remedy)**
Deshpande et al., 2022	Immunodaat capsules & Conventional management for COVID-19	37	40.54 ± 12.05	30	Insomnia	ISI	-1.29	-0.75	< 0.05	Improved sleep<br><br>**(Natural remedy)**
Lau et al., 2023	SIM01	232	49.30	189	Insomnia	PACSQ-14	58% of patients experienced insomnia alleviation	44% of patients experienced insomnia alleviation	< 0.05	Improved sleep<br><br>**(Natural remedy)**
Kang et al., 2023	Aromatherapy	30	31.30 ± 11.03	4	Sleep quality	KMLSEQ	+261.26	+112.54	0.089	Did not significantly improve sleep<br><br>**(Natural remedy)**
An et al., 2022	Shugan Jieyu capsule	99	54.5 ± 10.8	41	Insomnia	ISI	-11.2	-2.6	<0.001	Improved sleep<br><br>**(Natural remedy - enhancing liver function)**
Li et al., 2021	Shumian capsule	96	55	14	TCM symptom of insomnia	VAS	-3.1	-2.1	0.001	Improved sleep<br><br>**(Natural remedy - enhancing liver function)**
Yang et al., 2023	Xiaoyao capsule	96	54	14	Self-assessed sleep quality	VAS	-3.4	-3.3	>0.05	Did not significantly improve sleep<br><br>**(Natural remedy - enhancing liver function)**
Mosavi et al., 2023	Mindfulness-based stress reduction (MBSR)<br><br>Aerobic exercise<br><br>Combination of MBSR and aerobic exercise	MBSR - 15<br><br>Aerobic exercise - 15<br><br>Combination of MBSR and aerobic exercise - 15	NR	14	Sleep quality	PSQI	NR	NR	0.001	Combination of MBSR and aerobic exercise improved sleep the most<br><br>**(Exercise with mindfulness training)**
Hausswirth et al., 2023	Neuro-Mediation Program	17	47.10 ± 8.30	10	Sleep quality	SSQ	+3.4	+2.0	< 0.05	Improved sleep<br><br>**(Stimulation therapy with mindfulness training)**

Table S3

Study	Intervention	Reporting of adverse event	Details of adverse event
Hajibashi et al., 2023	Pulmonary telerehabilitation & Progressive muscle relaxation	NR	NR
Keskin and Saka, 2023	Strengthening exercises & Relaxation exercises	NR	NR
Hausswirth et al., 2023	Neuro-Mediation Program	NR	NR
Kang et al., 2023	Aromatherapy	NR	NR
Putilina et al., 2021	Cytoflavin tablets	None observed	N.A.
Tartibian et al., 2022	Home-based moderate-intensity continuous training (MICT), resistance training (RT), and Combined aerobic and resistance training (CET)	None observed	N.A.
Mosavi et al., 2023	Mindfulness-based stress reduction, aerobic exercise, and combination of mindfulness-based stress reduction and aerobic exercise	None observed	N.A.
Li et al., 2021	Shumian capsule	None observed	N.A.
Tanashyan et al., 2022	Brainmax	Observed	Transient, did not require discontinuation of therapy.
Zilberman-Itskovich et al., 2022	Hyperbaric oxygen therapy	Observed	13 adverse events reported:<br>• Barotrauma (HBOT: 4; Control: 3)<br>• Ear pain (HBOT: 1)<br>• Palpitation (HBOT: 3; Control: 1)<br>• Allergic rash (Control: 1)<br>• Headache (HBOT: 1)<br>• Chest or epigastric pain (HBOT: 1; Control: 2)<br>• Fever (HBOT: 1; Control: 1)<br>• Urinary tract infection (Control: 2)<br>• Herpes zoster infection (HBOT: 1)<br>• Cellulitis (Control: 1)<br>• Pre-syncope (Control: 1)<br><br>No patients discontinued treatment due to side effects.
An et al., 2022	Shugan Jieyu capsule	Observed	5 adverse events reported:<br>• Mild diarrhoea<br>• Abnormal liver function<br>• Cervical polyp<br>• Stomach pain<br>• Mild gastrointestinal discomfort)<br><br>Mild and did not lead to discontinuation of treatment.
Deshpande et al., 2022	Immunodaat capsules & Conventional COVID-19 management	Observed	Minor adverse events<br>• Abdominal discomfort<br>• Distension<br><br>Resolved on their own without pausing treatment.
Yang et al., 2023	Xiaoyao capsule	Observed	1% in experimental group, 2.1% in control group. No significant difference (P > 0.05).
Lau et al., 2023	SIM01	Observed	10% in experimental group, 11% in control group. No significant difference (p=0.63).<br><br>5 adverse events reported:<br>• Common gastrointestinal adverse events included diarrhea (SIM01: 2; Placebo: 5)<br>• Bloating (SIM01: 1; Placebo: 2)<br>• Epigastric pain (SIM01: 1; Placebo: 1)<br>• Abdominal pain (SIM01: 1; Placebo: 0)<br>• Flatulence (SIM01: 1; Placebo: 0).<br><br>Mild and self-limiting.

Table S4

Study	Risk of bias	Inconsistency	Indirectness	Imprecision	Publication bias	Quality of evidence
Hajibashi et al., 2023	Low	Not serious	Not serious	Serious	Undetected	Moderate
Keskin and Saka, 2023	Some concerns	Not serious	Not serious	Serious	Undetected	Low
Tanashyan et al., 2022	Low	Not serious	Not serious	Less serious	Undetected	Moderate
Zilberman-Itskovich et al., 2022	Low	Not serious	Not serious	Serious	Undetected	Moderate
Putilina et al., 2021	Some concerns	Not serious	Not serious	Potential imprecision	Undetected	Low
Tartibian et al., 2022	High	Not serious	Not serious	Less serious	Undetected	Low
Mosavi et al., 2023	High	Not serious	Not serious	Serious	Undetected	Low
An et al., 2022	Low	Not serious	Not serious	Potential imprecision	Undetected	Moderate
Deshpande et al., 2022	Some concerns	Not serious	Not serious	Serious	Undetected	Low
Li et al., 2021	High	Not serious	Not serious	Potential imprecision	Undetected	Low
Yang et al., 2023	High	Not serious	Not serious	Potential imprecision	Undetected	Low
Hausswirth et al., 2023	Some concerns	Not serious	Not serious	Serious	Undetected	Low
Lau et al., 2023	Low	Not serious	Not serious	Less serious	Undetected	Moderate
Kang et al., 2023	Some concerns	Not serious	Not serious	Serious	Undetected	Low

Table S5

Embase

No.	Searches
#1	exp sleep/
#2	exp sleep disturbance/
#3	exp sleep disorder/
#4	sleep*.ab,kw,ti
#5	(sleepless* or wakeful* or vigilan* or watchful* or awake* or restless* or inability to sleep).ab,kw,ti.
#6	fall* asleep.ab,kw,ti.
#7	stay* asleep.ab,kw,ti.
#8	asleep*.ab,kw,ti
#9	poor sleep*.ab,kw,ti.
#10	quality sleep.ab,kw,ti
#11	(insomnia or insomnolence or parasomnia).ab,kw,ti.
#12	#1 or #2 or #3 or #4 or #5 or #6 or #7 or #8 or #9 or #10 or #11
#13	exp long COVID/
#14	covid sequelae.ab,kw,ti.
#15	(long coronavirus disease 2019 or post coronavirus disease 2019 or after coronavirus disease 2019).ab,kw,ti.
#16	(long severe acute respiratory syndrome coronavirus 2 or post severe acute respiratory syndrome coronavirus 2 or after severe acute respiratory syndrome coronavirus 2).ab,kw,ti.
#17	(long SARS Cov 2 or post SARS Cov 2 or after SARS Cov 2).ab,kw,ti.
#18	(long COVID* or long-COVID* or post COVID* or post-COVID* or long-haul COVID* or after COVID* or post-COVID* conditions or post-acute COVID* or post-infectious COVID*).ab,kw,ti.
#19	#13 or #14 or #15 or #16 or #17 or #18
#20	random*.ab,kw,ti.
#21	control* clinical trial*.ab,kw,ti.
#22	control* stud*.ab,kw,ti.
#23	crossover*.ab,kw,ti.
#24	group*.ab,kw,ti.
#25	#20 or #21 or #22 or #23 or #24
#26	#12 and #19 and #25

Pubmed

((sleep) OR (sleep disturbance) OR (sleep disorder) OR (sleep*[Title/Abstract]) OR (sleepless*[Title/Abstract]) OR (wakeful*[Title/Abstract]) OR (vigilan*[Title/Abstract]) OR (watchful*[Title/Abstract]) OR (awake*[Title/Abstract]) OR (restless*[Title/Abstract]) OR (inability to sleep[Title/Abstract]) OR (fall* asleep[Title/Abstract]) OR (stay* asleep[Title/Abstract]) OR (asleep*[Title/Abstract]) OR (poor sleep*[Title/Abstract]) OR (quality sleep[Title/Abstract]) OR (insomnia[Title/Abstract]) OR (insomnolence[Title/Abstract]) OR (parasomnia[Title/Abstract])) AND ((long COVID) OR (covid sequelae[Title/Abstract]) OR (long coronavirus disease 2019[Title/Abstract]) OR (post coronavirus disease 2019[Title/Abstract]) OR (after coronavirus disease 2019[Title/Abstract]) OR (long severe acute respiratory syndrome coronavirus 2[Title/Abstract]) OR (post severe acute respiratory syndrome coronavirus 2[Title/Abstract]) OR (after severe acute respiratory syndrome coronavirus 2[Title/Abstract]) OR (long SARS Cov 2[Title/Abstract]) OR (post SARS Cov 2[Title/Abstract]) OR (after SARS Cov 2[Title/Abstract]) OR (long COVID*[Title/Abstract]) OR (long-COVID*[Title/Abstract]) OR (post COVID*[Title/Abstract]) OR (post-COVID*[Title/Abstract]) OR (long-haul COVID*[Title/Abstract]) OR (after COVID*[Title/Abstract]) OR (post-COVID* conditions[Title/Abstract]) OR (post-acute COVID*[Title/Abstract]) OR (post-infectious COVID*[Title/Abstract])) AND (random*[Title/Abstract] OR control* clinical trial*[Title/Abstract] OR control* stud*[Title/Abstract] OR crossover*[Title/Abstract] OR group*[Title/Abstract])

Web of Science

	Topic	sleep* or sleep disturbance* or sleep disorder* or sleepless* or wakeful* or awake* or restless* or inability to sleep or fall* asleep or stay* asleep or asleep* or poor sleep* or quality sleep* or insomnia or insomnolence or parasomnia
And	Topic	long COVID* or long-COVID* or post COVID* or post-COVID* or long-haul COVID* or after COVID* or post-COVID* conditions or post-acute COVID* or post-infectious COVID* or covid sequelae or long coronavirus disease 2019 or post coronavirus disease 2019 or after coronavirus disease 2019 or long severe acute respiratory syndrome coronavirus 2 or post severe acute respiratory syndrome coronavirus 2 or after severe acute respiratory syndrome coronavirus 2 or long SARS Cov 2 or post SARS Cov 2 or after SARS Cov 2
And	Topic	random* or control* clinical stud* or control* stud* or crossover* or group*

Cochrane Library

	Title Abstract Keyword	sleep* or sleep disturbance* or sleep disorder* or sleepless* or wakeful* or awake* or restless* or inability to sleep or fall* asleep or stay* asleep or asleep* or poor sleep* or quality sleep* or insomnia or insomnolence or parasomnia
And	Title Abstract Keyword	long COVID* or long-COVID* or post COVID* or post-COVID* or long-haul COVID* or after COVID* or post-COVID* conditions or post-acute COVID* or post-infectious COVID* or covid sequelae or long coronavirus disease 2019 or post coronavirus disease 2019 or after coronavirus disease 2019 or long severe acute respiratory syndrome coronavirus 2 or post severe acute respiratory syndrome coronavirus 2 or after severe acute respiratory syndrome coronavirus 2 or long SARS Cov 2 or post SARS Cov 2 or after SARS Cov 2
And	Title Abstract Keyword	random* or control* clinical stud* or control* stud* or crossover* or group*

China National Knowledge Infrastructure

SU%=('失眠' or '失眠症' or '不眠症' or '不寐' or '嗜睡' or '睡眠' or '睡觉') and SU%=('新冠病毒' or '长期新冠病毒' or '新冠' or '长期新冠' or '新冠后遗症')

Wanfang Data

主题:('失眠' or '失眠症' or '不眠症' or '不寐' or '嗜睡' or '睡眠' or '睡觉') and 主题:('新冠病毒' or '长期新冠病毒' or '新冠' or '长期新冠' or '新冠后遗症')

Table S6

Study	IG Intervention	Details of intervention
Tanashyan et al., 2022	Brainmax<br>(succinic acid coordination complex with trimethylhydrazinium)	**Target cellular oxygen metabolism**<br><br>Succinic acid complex<br>•Boosts mitochondrial respiration.<br>•Promotes regular cellular functions and modulates metabolism during stress, preserving cellular vitality.<br><br>Trimethylhydrazinium component of the complex<br>•Shifts cells into anaerobic metabolism, decreasing oxygen availability for succinate oxidation. This normalizes energy exchange in cells, even under hypoxic conditions, preventing the formation of harmful free radicals and mitigating the effects of ischemic-hypoxic tissue damage.
Putilina et al., 2021	Cytoflavin tablets	**Target cellular oxygen metabolism**<br><br>•Alleviates asthenia and correct cognitive impairments by reducing inflammation, oxidative stress, and hypoxia.<br>•Enhances cellular energy production, reduces free radical production, restores antioxidant defenses, and improves mitochondrial function, facilitating glucose and fatty acid utilization.

Table S7

Study	IG Intervention	Details of intervention
Hajibashi et al., 2023	Pulmonary telerehabilitation<br>(Breathing exercises + Strength training) & Progressive muscle relaxation<br>(muscle contraction and relaxation)	**Therapeutic exercise**<br><br>Progressive muscle relaxation (PMR)<br>• Reduces anxiety and improves sleep quality.<br>• Reduces fatigue and depression and Improves sleep quality in chronic obstructive pulmonary disease (COPD) patients.<br><br>Pulmonary telerehabilitation (PTR)<br>• Enhances functional capacity, quality of life, and alleviates anxiety.
Keskin and Saka, 2023	Strengthening exercises<br>(weights resistance exercises) &<br>Relaxation exercises<br>(gradual muscle contraction and relaxation)	**Therapeutic exercise**<br><br>• Reduces pain.<br>• Enhances sleep quality (attributed to factors like exposure to chronobiotic light post-isolation, increased physical activity, and psychosocial engagement), cognitive function, and physical ability in painful conditions.
Tartibian et al., 2022	Home-based moderate-intensity continuous training (MICT) - Progressive low- to moderate-intensity walking<br><br>Home-based Resistance training (RT) - Lower-body resistance exercises + Upper-body resistance exercises<br><br>Home-based Combined aerobic and resistance training (CET) - MICT followed by RT	**Therapeutic exercise**<br><br>Regular physical activity enhances immune function, reduce systemic inflammation, and improve various health conditions.<br>• Improvements in creatine kinase (CK), lactate dehydrogenase (LDH), C-reactive protein (CRP), troponin, d-dimer, creatinine, urea, potassium, sodium, white blood cells (WBC), lymphocytes, red blood cells (RBC), platelets, hemoglobin, and hematocrit markers.<br>• Suggest positive effects on recovery and cardiovascular health, as well as renal function recovery and blood oxygen-carrying capacity.<br>• CET was found to be particularly effective.
Zilberman-Itskovich et al., 2022	Hyperbaric oxygen therapy<br>(breathing 100% oxygen)	**Natural remedy**<br><br>Exploits the "hyperoxic-hypoxic paradox" to induce neuroplasticity and aid brain recovery by activating gene expression and metabolic pathways crucial for regulation without harmful hypoxia.<br>• Improves cognitive, psychiatric, fatigue, sleep, and pain symptoms in post-COVID-19 patients, associated with increased brain perfusion and microstructural changes in key regions.<br>• Specifically improves dysexecutive functions, alleviates psychiatric symptoms (depression, somatization), reduces pain interference, and mitigates fatigue.<br>• The treatment's efficacy parallels its success in fibromyalgia and chronic fatigue syndrome, suggesting that it likely operates through immune response modulation, angiogenesis, mitochondrial restoration, and neurogenesis.
Deshpande et al., 2022	Immunodaat capsules (Elderberry extract) & Conventional management for COVID-19	**Natural remedy**<br><br>Elderberry<br>• Rich in phytonutrients, vitamins, minerals, and essential oils<br>• Has immune-boosting properties in preventing and managing respiratory ailments. Its flavonoids stimulate cytokine production by monocytes, bolstering the immune system.<br>• Inhibits influenza virus hemagglutination, impeding virus-cell receptor adhesion.<br>• The anthocyanins in elderberry possess potent anti-inflammatory effects akin to acetylsalicylic acid, potentially explaining the observed reductions in inflammation, pain, and fever.
Lau et al., 2023	SIM01<br>(synbiotic preparation containing B adolescentis, Bifidobacterium bifidum, and Bifidobacterium longum strains with galacto-oligosaccharides, xylo-oligosaccharides, and resistant dextrin)	**Natural remedy**<br><br>The research suggests the potential of gut microbiome-targeted therapies for PACS in the post-COVID era.<br><br>SIM01 → A therapeutic targeting gut dysbiosis which may modify the immune response and improve symptoms<br>• Improvements in insomnia, increased gut bacterial diversity, more short-chain acid-producing bacteria, and fewer antimicrobial resistant genes.<br>• Prebiotic compounds in SIM01 enhanced gut microbiota by increasing beneficial bacteria like Bifidobacterium pseudocatenulatum and Bifidobacterium longum & suppressing pathogenic bacteria like Klebsiella pneumoniae and Parabacteroides merdae.<br>• Relieved gastrointestinal upset, similar to post-infectious irritable bowel syndrome (PI-IBS) symptoms following SARS-CoV-2 infection.<br>• Emerges as a safe and promising treatment for PACS.
Kang et al., 2023	Aromatherapy<br>(Inhalation of aroma essential oil consisting of lavender and ylang-ylang, mixed at a ratio of 8:2)	**Natural remedy**<br><br>Lavender<br>• Has a high linalyl acetate content.<br>• Recommended for respiratory issues like bronchitis and colds.<br>• Has calming effects on the nervous system.<br><br>Ylang ylang<br>• Contain benzyl acetate.<br>• Offers calming properties.
An et al., 2022	Shugan Jieyu capsule<br>(Guanyelianqiao + Ciwujia)	**Target poor sleep with anti-depression and calming effects through enhancing liver function**<br><br>Gyanyelianqiao (*Herba Hyperici Perforati*, GYLQ)<br>• Cold constitution in nature with a pungent in taste.<br>• Soothes the liver.<br>• Reduces depression.<br>• Has calming effects.<br>• Reduces swelling.<br>• Promotes lactation.<br>• Activates the central nervous systems by modulating neurotransmitter levels and adrenergic receptor density.<br>• Regulates the thalamus-pituitary-adrenal axis.<br><br>Ciwujia (*Radix et Caulis Acanthopanacis Santicosi*, CWJ)<br>• Heat constitution in nature with a pungent, slightly bitter taste.<br>• Contains non-aromatic unsaturated organic acids and a variety of trace minerals.<br>• Supports spleen, kidney and heart function<br>• Invigorates Qi, strengthening the kidney.<br>• Has calming effects.<br>• Acts as a sedative and anti-fatigue agents, aiding in establishing normal sleep cycles and treating sleep disorders.<br>• Its efficacy is comparable to Fluoxetine (western medicine), with fewer adverse drug reactions and greater safety.
Li et al., 2021	Shumian capsule<br>(Suanzaoren + Chaihu + Baishao + Jiangcan + Dengxincao)	**Target poor sleep with anti-depression and calming effects through enhancing liver function**<br><br>Shumian capsule has been on the market for more than 10 years, has been widely used in patients with insomnia.<br><br>Suanzaoren (Semen Zizyphi Spinosae)<br>• Tonify the liver and calm the heart, astringing sweat, and promoting the secretion of saliva.<br><br>Chaihu (Radix Bupleuri)<br>• Can relieve fever, soothe the liver, and elevate Yang Qi.<br><br>Baishao (Radix Albus Paeoniae Lactiflorae) combined with Hehuanhua (Flos Albiziae) and Hehuanpi (Cortex Albizziae Julibrissinis)<br>• Soothe the liver, relieve depression, and calm the mind.<br><br>Jiangcan (Stiff Silkworm) and Chantui (Ps Cicadae)<br>• Soothe the liver and relieve spasms.<br><br>Dengxincao (Juncaceae Juss)<br>• Soothe the liver, relieve depression, and calm the mind.<br><br>Research indicates that combining Shumian capsules with psychological intervention enhances sleep quality and alleviates anxiety and depression in patients.
Yang et al., 2023	Xiaoyao capsule	**Target poor sleep with anti-depression and calming effects through enhancing liver function**<br><br>Xiaoyao capsule is formulated from Xiaoyao San (逍遥散), comprising Chaihu (Radix Bupleuri Chinensis), Danggui (Radix Angelicae Sinensis), Baishao (Radix Paeoniae Alba), Baizhu (Rhizoma Atractylodis Macrocephalae), Fuling (Poria), Shengjiang (Rhizoma Zingiberis Recens), Bohe (Herba Menthae Haplocalycis), and Zhigancao (Radix Glycyrrhizae).<br><br>It functions to<br>• Clear liver stagnation<br>• Strengthen the spleen<br>• Enrich blood<br>• Regulate menstruation
Mosavi et al., 2023	Mindfulness-based stress reduction (MBSR)<br><br>Aerobic exercise<br><br>Combination of MBSR and aerobic exercise	**Exercise with lifestyle modification**<br><br>Aerobic exercise<br>• Promotes the release of neurotrophins, crucial for brain development and are linked to improved sleep, reduced neuropathic pain, and psychosis.<br>• Reduces stress and enhances the release of neurotransmitters, aiding in sleep quality and initiation.<br>• Mitigates mental rumination, a major contributor to sleep disorders.<br><br>Mindfulness practices<br>• Enhances sleep quality.
Hausswirth et al., 2023	Neuro-Mediation Program<br>(non-invasive cognitive stimulation and mindfulness training with sound therapy and light stimulations)	**Stimulation therapy with mindfulness training**<br><br>• Significant reductions in physical and mental fatigue, muscle and joint pain, symptoms of depression and anxiety, and mood disturbances, alongside significant enhancements in sleep quality.

PRISMA 2020 Checklist

Section and Topic	Item #	Checklist item	Location where item is reported
**TITLE**			
Title	1	Identify the report as a systematic review.	Page 1
**ABSTRACT**			
Abstract	2	See the PRISMA 2020 for Abstracts checklist.	Page 2
**INTRODUCTION**			
Rationale	3	Describe the rationale for the review in the context of existing knowledge.	Page 2, 3; Section 1
Objectives	4	Provide an explicit statement of the objective(s) or question(s) the review addresses.	Page 3; Section 1
**METHODS**			
Eligibility criteria	5	Specify the inclusion and exclusion criteria for the review and how studies were grouped for the syntheses.	Page 3; Section 2.1
Information sources	6	Specify all databases, registers, websites, organisations, reference lists and other sources searched or consulted to identify studies. Specify the date when each source was last searched or consulted.	Page 3; Section 2.1
Search strategy	7	Present the full search strategies for all databases, registers and websites, including any filters and limits used.	Supplementary table 5
Selection process	8	Specify the methods used to decide whether a study met the inclusion criteria of the review, including how many reviewers screened each record and each report retrieved, whether they worked independently, and if applicable, details of automation tools used in the process.	Page 3; Section 2.1
Data collection process	9	Specify the methods used to collect data from reports, including how many reviewers collected data from each report, whether they worked independently, any processes for obtaining or confirming data from study investigators, and if applicable, details of automation tools used in the process.	Page 3; Section 2.2
Data items	10a	List and define all outcomes for which data were sought. Specify whether all results that were compatible with each outcome domain in each study were sought (e.g. for all measures, time points, analyses), and if not, the methods used to decide which results to collect.	Page 4; Section 2.3
	10b	List and define all other variables for which data were sought (e.g. participant and intervention characteristics, funding sources). Describe any assumptions made about any missing or unclear information.	Page 4; Section 2.3
Study risk of bias assessment	11	Specify the methods used to assess risk of bias in the included studies, including details of the tool(s) used, how many reviewers assessed each study and whether they worked independently, and if applicable, details of automation tools used in the process.	Page 4; Section 2.4
Effect measures	12	Specify for each outcome the effect measure(s) (e.g. risk ratio, mean difference) used in the synthesis or presentation of results.	Page 4; Section 2.3
Synthesis methods	13a	Describe the processes used to decide which studies were eligible for each synthesis (e.g. tabulating the study intervention characteristics and comparing against the planned groups for each synthesis (item #5)).	Page 4; Section 2.3
	13b	Describe any methods required to prepare the data for presentation or synthesis, such as handling of missing summary statistics, or data conversions.	Page 4; Section 2.3, 2.4, 2.5
	13c	Describe any methods used to tabulate or visually display results of individual studies and syntheses.	Page 4; Section 2.3
	13d	Describe any methods used to synthesize results and provide a rationale for the choice(s). If meta-analysis was performed, describe the model(s), method(s) to identify the presence and extent of statistical heterogeneity, and software package(s) used.	Page 4; Section 2.3

PRISMA 2020 Checklist

Section and Topic	Item #	Checklist item	Location where item is reported
	13e	Describe any methods used to explore possible causes of heterogeneity among study results (e.g. subgroup analysis, meta-regression).	Page 4; Section 2.3
	13f	Describe any sensitivity analyses conducted to assess robustness of the synthesized results.	Page 4; Section 2.3
Reporting bias assessment	14	Describe any methods used to assess risk of bias due to missing results in a synthesis (arising from reporting biases).	Page 4; Section 2.4
Certainty assessment	15	Describe any methods used to assess certainty (or confidence) in the body of evidence for an outcome.	Page 4; Section 2.5
**RESULTS**			
Study selection	16a	Describe the results of the search and selection process, from the number of records identified in the search to the number of studies included in the review, ideally using a flow diagram.	Page 4; Section 3.1
	16b	Cite studies that might appear to meet the inclusion criteria, but which were excluded, and explain why they were excluded.	Page 4; Section 3.1
Study characteristics	17	Cite each included study and present its characteristics.	Page 5; Section 3.2
Risk of bias in studies	18	Present assessments of risk of bias for each included study.	Page 9; Section 3.7
Results of individual studies	19	For all outcomes, present, for each study: (a) summary statistics for each group (where appropriate) and (b) an effect estimate and its precision (e.g. confidence/credible interval), ideally using structured tables or plots.	Page 7-8; Section 3.3, 3.4
Results of syntheses	20a	For each synthesis, briefly summarise the characteristics and risk of bias among contributing studies.	Page 9; Section 3.7
	20b	Present results of all statistical syntheses conducted. If meta-analysis was done, present for each the summary estimate and its precision (e.g. confidence/credible interval) and measures of statistical heterogeneity. If comparing groups, describe the direction of the effect.	Page 9; Section 3.5
	20c	Present results of all investigations of possible causes of heterogeneity among study results.	Page 9; Section 3.5
	20d	Present results of all sensitivity analyses conducted to assess the robustness of the synthesized results.	n/a
Reporting biases	21	Present assessments of risk of bias due to missing results (arising from reporting biases) for each synthesis assessed.	Page 9; Section 3.7
Certainty of evidence	22	Present assessments of certainty (or confidence) in the body of evidence for each outcome assessed.	Page 11; Section 3.8
**DISCUSSION**			
Discussion	23a	Provide a general interpretation of the results in the context of other evidence.	Page 11, 12; Section 4.1, 4.2
	23b	Discuss any limitations of the evidence included in the review.	Page 12; Section 4.3
	23c	Discuss any limitations of the review processes used.	Page 12;

Section and Topic	Item #	Checklist item	Location where item is reported
			Section 4.3
	23d	Discuss implications of the results for practice, policy, and future research.	Page 12; Section 4.4
**OTHER INFORMATION**			
Registration and protocol	24a	Provide registration information for the review, including register name and registration number, or state that the review was not registered.	Page 2; Abstract
	24b	Indicate where the review protocol can be accessed, or state that a protocol was not prepared.	Page 2; Abstract
	24c	Describe and explain any amendments to information provided at registration or in the protocol.	n/a
Support	25	Describe sources of financial or non-financial support for the review, and the role of the funders or sponsors in the review.	Page 13; Section 6
Competing interests	26	Declare any competing interests of review authors.	Page 13; Section 6
Availability of data, code and other materials	27	Report which of the following are publicly available and where they can be found: template data collection forms; data extracted from included studies; data used for all analyses; analytic code; any other materials used in the review.	Page 3; Section 2.1

From: Page MJ, McKenzie JE, Bossuyt PM, Boutron I, Hoffmann TC, Mulrow CD, et al. The PRISMA 2020 statement: an updated guideline for reporting systematic reviews. BMJ 2021;372:n71. doi: 10.1136/bmj.n71
